# Superlinear Photoluminescence by Ultrafast Laser Pulses in Dielectric Matrices with Metal Nanoclusters

**DOI:** 10.1038/s41598-019-42174-1

**Published:** 2019-04-05

**Authors:** J. Bornacelli, C. Torres-Torres, H. G. Silva-Pereyra, G. J. Labrada-Delgado, A. Crespo-Sosa, J. C. Cheang-Wong, A. Oliver

**Affiliations:** 1 Sección de Estudios de Posgrado e Investigación, Escuela Superior de Ingeniería Mecánica y Eléctrica Unidad Zacatenco, Instituto Politécnico Nacional, 07738 Ciudad de México, Mexico; 2 IPICyT, División de Materiales Avanzados, Camino a la presa San José 2055, San Luis Potosí, SLP 78216 Mexico; 3 Instituto de Física, Universidad Nacional Autónoma de México, 04510 Ciudad de México, Mexico

## Abstract

An intense photoluminescence emission was observed from noble metal nanoclusters (Pt, Ag or Au) embedded in sapphire plates, nucleated by MeV ion-implantation and assisted by an annealing process. In particular, the spectral photoluminescence characteristics, such as range and peak emission, were compared to the behavior observed from Pt nanoclusters embedded in a silica matrix and excited by UV irradiation. Correlation between emission energy, nanoclusters size and metal composition were analyzed by using the scaling energy relation *E*_*Fermi*_/*N*^1/3^ from the spherical Jellium model. The metal nanocluster luminescent spectra were numerically simulated and correctly fitted using the bulk Fermi energy for each metal and a Gaussian nanoclusters size distribution for the samples. Our results suggest protoplasmonics photoluminescence from metal nanoclusters free of surface state or strain effects at the nanoclusters-matrix interface that can influence over their optical properties. These metal nanoclusters present very promising optical features such as bright visible photoluminescence and photostability under strong picosecond laser excitations. Besides superlinear photoluminescence from metal nanoclusters were also observed under UV high power excitation showing a quadratic dependence on the pump power fluence.

## Introduction

Nanometals embedded in glasses have been studied in the last decades due to their unique optical properties mainly due to the presence of localized surface plasmon resonances (LSPR) in larger (size >2 nm) metal nanoparticles (NPs). Interesting optical effects such as enhanced nonlinear optical properties^1,2^, enhanced photoluminescence by plasmon-emitters coupling^3–5^ and application in sensing^6^ or optical storage^7^ have been demonstrated and rely on their local-electromagnetic field enhanced effects exhibited by larger plasmonic NPs. Metallic nanoparticles (such as Ag, Au, Pt, Cu) exhibit a particular optical response depending on their size, density, size distribution and shape^8–12^. The glass matrix is an excellent encapsulating medium, due to its wide range of optical transmission from UV to infrared depending on their purity and composition. Besides, the glass protects nanometals from oxidation, aggregation and environmental reaction. These versatile nanocomposites show stable optical responses that are attractive for long-term technological applications in nanoscale optoelectronics and photonics. In the absence of LSPR nanometals of ultra-small size also exhibit new and less studied optical properties such as photoluminescence^13–15^ (PL), magnetism^16,17^, molecular chirality^18,19^, and applications in light energy conversion^20^ and photosensitizers^21^ have also been demonstrated. Ultra-small nanometals or nanoclusters (NCs, size <2 nm) tend to become aggregated to form larger NPs and several approaches to stabilize them by organic ligand such as dendrimers^22^ or thiolated^20^ have been attempted. However, the presence of this ligand influences the optical properties of the nanocluster-ligand complex and its physical stability under laser irradiation is also affected^23^.

On the other hand, scarcely systematic investigation has been done to synthesize and understand the optical properties of sub-nanometer metal NCs embedded in dielectric inorganic matrices. Room temperature PL have been observed in Ag, Au and Pt NCs embedded in soda-lime silicate glasses by light synchrotron irradiation^24^, oxyfluoride and fluoroborated glasses by melt-quenching technique^25,26^ and in silica matrices by ion-implantation^27–29^. Among diverse techniques used for the fabrication of metal NPs in dielectrics, ion-implantation is very attractive because it allows the possibility of synthesizing metallic NPs embedded in the near-surface region of the substrate, controlling the depth and the concentration of the NPs. Moreover, ion-implantation is widely used today for the large-scale production of semiconductors and integrated circuits. The most common dielectric material used for chip fabrication is silicon oxide (SiO_2_), but also sapphire (Al_2_O_3_) matrices are frequently employed to make waveguides and with interesting uses as host materials for rare earth ions, such as Er, emitting in the telecommunication band range^30^. Indeed Ag NCs have been synthesized in fluorobate glass phosphor combined with Eu ions to obtain a solid state white light emitter^25^. Recently, direct applications of luminescent Au NCs embedded in silica to enhance luminescent emission of Er ions at telecommunications band have been demonstrated^28^. Non-linear optical properties have been also pointed out for Pt NCs embedded in silica matrix under UV excitation, exhibiting an ultrafast response that make them attractive for applications in high-speed optoelectronics applications^31^.

To understand the photophysics of metal NCs, a simple scaling energy relation, *E*_*Fermi*_/*N*^1/3^, where *E*_*Fermi*_ is the bulk metal Fermi energy and *N* the number of atoms in the clusters, is commonly used to provide an approximated description of the energy emission from Au NCs^32^ and it is derived from quantum confinement effects in the Jellium model approximation. However, a deviation from this relation is found in thiolated Au clusters and also its application for other metals NCs is not suitable for describing their optical transition^33^. For Au NCs embedded in a silica matrix a decrease in energy emission with the number *N* of atoms in the NCs has been reported as predicted by Jellium model; however, their energy emissions do not match using the Au bulk Fermi energy^28^. Deviation from this scaling relation can be attributed to the presence of surface state or strain effect that actively participate in the electronic transitions that give rise to PL emissions. Most research has been conducted to find a synthesis method which minimizes the surface state effect over the metal NCs emission properties in order to understand and tune their optical properties. A simple description of their photophysics allows for further exploration of the PL properties in this kind of samples and could achieve an in-depth understanding to fully realize their use in practical applications. Moreover, metal NCs can have different luminescent properties depending on the host matrices and a lot of research must be done in order to find valuable properties and optimal synthesis condition to meet particular applications.

With these motivations, in this work we report for the first time bright photoluminescence from noble metal NCs embedded in sapphire matrices synthesized by MeV ion-implantation technique. To the best of our knowledge only PL emission from larger Au NPs in sapphire has been reported^34^ but not for room temperature as this study demonstrates. Metal clustering process in sapphire allows the formation of sub-nanometers-sized photoluminescent clusters, as well as larger plasmonic particles even at high temperature annealing, as it has been pointed out before^35^. This annealing also allows the complete recovery of the sample from the implantation damage that can affect over the optical properties of the nanocomposite. By using three different metals, similar synthesis conditions and the same matrix host, we obtained three different luminescence peaks, associated with the corresponding variations in the color emission: green (Pt NCs), yellow-orange (Au NCs) and orange (Ag NCs). For comparison, we have also synthesized Pt NCs but embedded in silica to elucidate possible clusters-matrix interface effects on their optical properties. Interestingly, we find that, regardless of the metal NCs, their PL spectra can be correctly fitted by assuming a Gaussian NC size distribution and the simple energy relation, *E*_*Fermi*_/*N*^1/3^. These metal NCs present fascinating optical features such as an intense and superlinear PL, which scale quadratically with the laser pump fluence. The superlinear increase of PL intensity can be derived from biexciton emission^36,37^, uncorrelated electron-holes pairs^38,39^, or a two-photon excitation process^40^ and results are discussed considering theses possible effects. Sub-nanometer-sized light-emitting metal NCs embedded in an inorganic matrix by ion-implantation offers the possibility of implementing nanoscale light source for potential photonics and optoelectronics applications to satisfy the demand of continued miniaturization. Moreover, metal NCs in sapphire and silica were formed by using similar condition of synthesis, i.e. similar ion-implantation energies and annealing temperatures, and this can be addressed to combine them in the same sapphire matrix to obtain a solid state nano-source of white light emission.

## Results and Analisis

**Figure 1:**
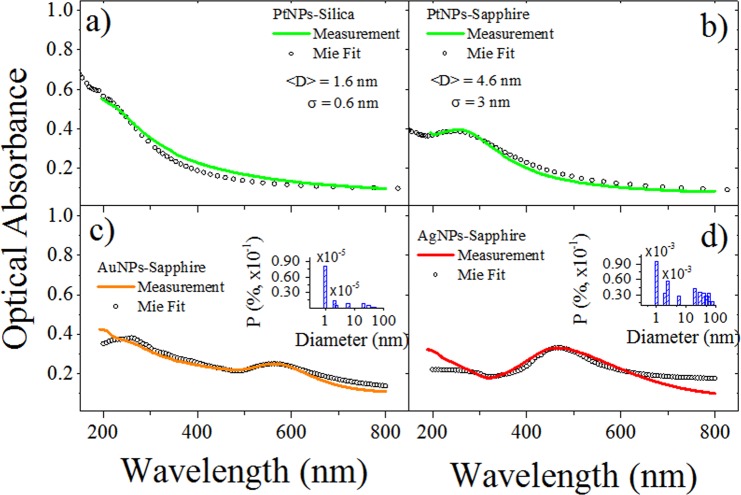
Optical absorption spectra for the different metallic NPs studied in this work: (**a**) Pt NPs embedded in silica; (**b**) Pt NPs in sapphire; (**c**) Au NPs in sapphire; (**d**) Ag NPs in sapphire. Inset graphs in (**c**) and (**d**) show the size distribution used to simulate the optical absorption according to Mie calculations for Au and Ag NPs in sapphire.

**Figure 2:**
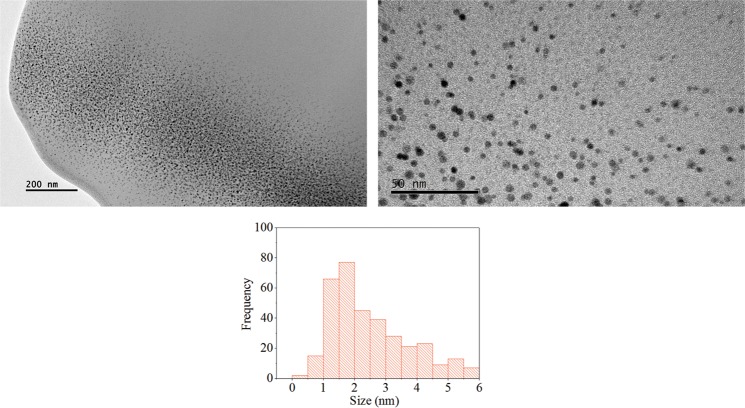
Panoramic cross-section (Left, scale bar: 200 nm) and a close-view (Right, scale bar: 50 nm) TEM micrographs of a sample with Pt particles embedded in silica. A representative histogram of the measured particle size distribution is included.

Figure 1 shows the optical absorption spectra measured from the different metal NPs (Ag, Au, Pt) embedded in sapphire and silica matrices, together with the Mie fitting of the peak and width of the resonance absorption band for each sample. In the case of the Au and Ag NCs in sapphire, the NC size distributions used to simulate the optical absorption spectra according to Mie calculations, are also shown^41,42^. Cross-section TEM micrograph in Fig. 2 was obtained from a similar prepared sample with Pt clusters in silica. In the representative image we can see the entire particle size distribution. The histogram in Fig. 2 shows a statistical count of diameters found in the micrograph, where the most abundant Pt NPs have a size between 1 nm and 2 nm of diameter. This average diameter agrees very well with that calculated by Mie fitting in Fig. 1 of about 1.6 nm. On the other hand, wider size distributions are observed for Au, Ag, and Pt particles in sapphire as estimated from Mie fitting in Fig. 1.

For Pt particles in sapphire the optical absorption can be numerically simulated using a Gaussian size distribution with a mean size of 4.6 nm and a standard deviation of 3 nm (65%). The presence of larger Pt NPs in sapphire results in a more pronounced plasmon absorption band as compared to Pt particles in silica. In contrast, for Ag and Au particles nucleated in sapphire their optical absorption spectra cannot be numerically simulated using a Gaussian size distribution. The size distributions employed for simulating the optical absorption spectra according to Mie calculations for Au and Ag NCs in sapphire are shown in the inset panel of Fig. 1(c,d). As it can be seen, the particle diameters range between 1 to 100 nm for the case of Ag implanted sample, and from 1 to 80 nm for Au implanted sample. This wider size distributions for Ag and Au NPs in sapphire have been also corroborated by our group by direct TEM observations^35^.

**Figure 3:**
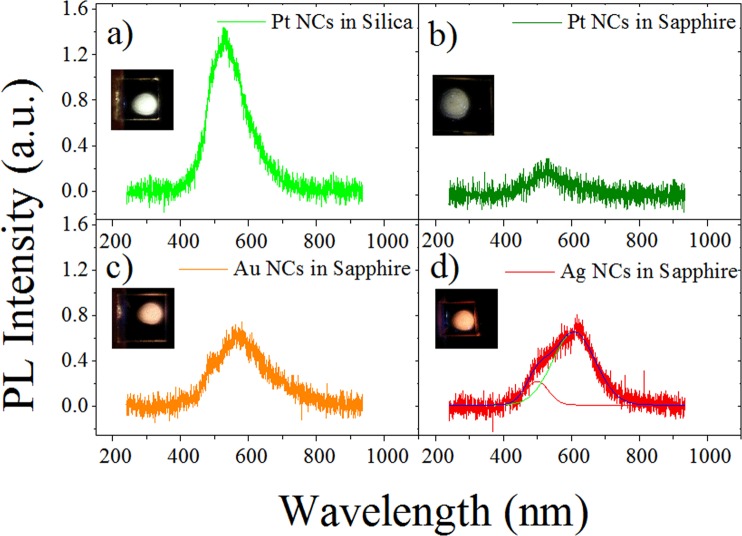
PL spectra of Pt, Au and Ag NCs embedded in silica (**a**) and sapphire (**b**–**d**) matrices. Laser excitation corresponds to 355 nm wavelength and a low pump fluence of 0.5 mJ/cm^2^.

PL spectra of the same set of samples with Ag, Au and Pt particles are shown in Fig. 3. Here we can notice that for Pt particles in silica or sapphire the spectrum is the same, except for the difference in intensity, with a peak centered at 530 nm and FWHM of about 140 nm. The PL emission from Pt particles in silica or sapphire comes from ultrasmall particles with size less than two nanometers^27,41^ Then, the resemblances in experimental spectral PL emission for Pt particles in silica or sapphire suggest a similar size distribution for ultrasmall Pt particles in both matrices; regardless of the presence of larger plasmonic NPs as predicted by Mie fit analysis.

On the other hand, Ag and Au particles in sapphire exhibit a more intense PL than that observed for Pt particles in sapphire. At first sight the PL spectra for Ag and Au particles in sapphire seem similar, with the main PL peak centered at 565 nm (FWHM ~ 200 nm) for Au NPs. However, the PL spectrum for the Ag particles exhibits two peaks, as shown by the deconvoluted curves in Fig. 3(d): the most intense PL peak is centered at 608 nm (FWHM ~160 nm) and the other one at 500 nm (FWHM ~100 nm). Emission peak wavelengths determine the color displayed by the glass by PL upon laser excitation: for Pt particles, green color is displayed, as shown inset photo in Fig. 3(a,b). For Au and Ag particles, yellow-orange and orange luminescence can be seen in the inset photos of Fig. 3(c,d), respectively. At this point it seems plausible to envisage a potential application in the development of nanoscale white light emitters by combining different metal NCs in the same sapphire matrix. Taking into account that annealing temperatures used for the different experiments were similar (∼1000 °C), it is possible considering to carry out a co-implantation of Ag and Pt ions in the same region within a sapphire matrix and nucleate NCs of both metals by applying a single thermal treatment.

By inspection of the optical absorptions and PL intensities of each sample, we can see that the sample with the lower plasmon absortion band (Pt in silica) exhibit the highest PL intensity as compared to Ag, Au, and Pt particles in sapphire. In fact, the PL intensity for Pt particles in silica is sixfold larger than the one in sapphire and two fold larger in comparison to the PL intensity of Ag and Au particles. The plasmon absorption band is related to the presence of bigger plasmonics metal NPs. A larger number of plasmonic NPs results in a more intense plasmon absortion. As the ion-implanted fluence is the same for all the samples studied in this work, then it can be assumed that they have a similar amount of metal ions per square centimeter. This means that for a sample exhibiting large plasmonic NPs there will be a smaller amount of available metal atoms to form subnanometer-sized particles or NCs. In consequence, if large plasmonic NPs are nucleated in the samples, a smaller amount of NCs is expected. Metal NCs can not support plasmon absorption, but they can absorb and emit light through HOMO-LUMO gap giving rise to PL emission^23^. In general, the PL intensity can be directly related to the number of metal nanoemitters in each sample and their internal quantum efficiency. In the case of Pt NCs, even though that the ion-implanted fluences are similar for both matrices, the thermal treatment in sapphire results in the nucleation of a larger number of plasmonic NPs, as it is evident from the comparison of the plasmon absorption bands in Fig. 1(a,b). Pt NCs in both dielectric matrices present similar spectral characteristics except for the PL intensities which can be related to the number of NCs formed in each sample after the thermal annealing. Similar PL spectra in both matrices suggest that absorption and emission processes in Pt NCs are not affected by surface states or strain phenomena at Pt NCs-matrix interface. Then similar internal quantum efficiency of Pt nanoemitters in both matrices it is expected; and the observed differences in PL intensity can be explained as a result of different number of nucleated Pt NCs.

Light emission from metal NCs depend on the particle size because HOMO-LUMO gap increases as the NC diameter decreases in size^32,43^. Derived from the Jellium model, an energy dependence on the number of atoms (*N*) was demonstrated for Au NCs. According to this simple model, the band gap of a cluster with *N* atoms will be given by a simple scaling relation, *E*_*Fermi*_/*N*^1/3^, where *E*_*Fermi*_ is the metal bulk Fermi energy. For a spherical cluster, its radius *R* is equal to *r*_*w*_*N*^1/3^, where *r*_*w*_ is the Wigner-Seitz radius^23^. PL emission energy can be assumed to be proportional to the band gap energy. Bulk Fermi energy data of Ag, Au and Pt are: 5.49, 5.53, 8.77^23,44^ eV, respectively, and their corresponding Wigner-Seitz radius are: 0.166, 0.165 and 0.160 nm^45^. By a simple inspection of the scaling energy relation, we see that for cluster size with similar number of atoms their energy emission increases mainly with the metal bulk Fermi energy. The bulk Fermi energy for Pt is larger than the corresponding value for Ag and Au. As we can observe from Fig. 3, the Pt NCs emission spectrum is peaked at shorter wavelength (green) than those from Ag or Au NCs (orange and yellow-orange, respectively), as expected from the spherical Jellium model.

We can estimate the cluster size range that gives rise photoluminescence emissions in the spectral range depicted in Fig. 3. The estimated data are shown in Table 1. The calculated size range agrees with the one estimated by fitting optical absorption of the samples using the Mie model and direct TEM observations for the case of Pt particles in silica. The experimental PL spectrum of Pt NCs ranges from 400–700 nm; this is in good agreement with Jellium model calculations for NCs size ranging between 0.9–1.6 nm of diameter and emitting light at these wavelengths. For the PL emission peak from sample with Pt NCs (530 nm) the corresponding NCs size is about 1.2 nm (*N* ∼ 53 Pt atoms). In the case of Ag and Au NCs their spectral range is due to NC sizes in the 0.6–1.1 nm, and 0.6–1.2 nm ranges, according to Jellium model, respectively. Ag NCs in sapphire exhibit two PL peaks at 500 and 608 nm which corresponds to the presence of NCs size of 0.74 nm (*N* ∼ 11 Ag atoms) and 0.9 nm (*N* ∼ 20 Ag atoms), respectively. For Au NCs their PL peak (565 nm) is due to the presence of NCs with a size of 0.84 nm (*N* ∼ 17 atoms).

**Table 1:** Estimated NCs diameters based on Jellium model and spectral emission characteristic of metal NCs embedded in silica or sapphire.

Metal NCs	Cluster Size Range (nm)	PL range (nm)	Cluster Size at peak emission (nm)	PL peak (nm)
Pt	0.9–1.6	400–700	1.2	530
Ag	0.6–1.1	408–680	0.9, 0.74^*^	608, 500^*^
Au	0.6–1.2	408–800	0.84	565

Asterisk values (*) are reported for deconvoluted PL peak observed in Fig. 3(d) for sample with AgNCs in sapphire.

**Figure 4:**
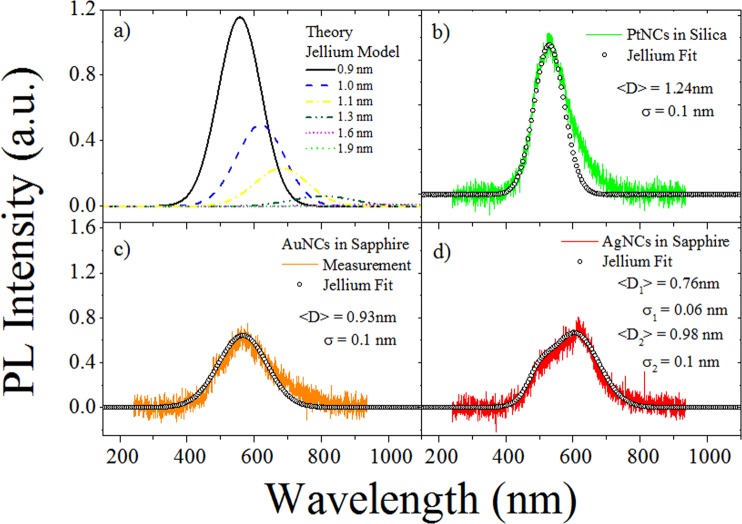
(**a**) Theoretical calculations by using the scaling relation *E*_*Fermi*_/*N*^1/3^ and several Gaussian size distributions to compute the PL emission energies of metal AuNCs. (**b**–**d**) Show the same theoretical fitting using the scaling energy relation from the Jellium model and a Gaussian size distribution for the Pt, Au and Ag NCs. Mean size and standard deviation are also shown for each sample.

To compute a general expression for the PL spectra, we can assume a Gaussian size distribution associated with the metal NCs. It can be considered that the number of atoms is proportional to the volume of the NCs, and for larger NCs more carriers take part in optical transition. Then the probability of PL emission is proportional to the volume, *d*^3^ (*d* is the diameter) of the metal NCs^46,47^. Oscillator strength can be assumed to vary inversely proportional with the NCs size, *f* = *d*^−*γ*^, with *γ* an adimensional constant whose exact value has to be adjusted in order to reproduce the experimental PL spectrum. For semiconductor quantum dots, *γ* takes values comprended between 1 and 6^46–49^. The probability for PL emission is then given by^50^:

1 $$P(d)\propto {d}^{-\gamma }{d}^{3}{e}^{-[\frac{{(d-\langle D\rangle )}^{2}}{2{\sigma }^{2}}]}$$

where 〈*D*〉 is the mean size of the NCs and *σ* is the standard deviation. By using the scaling energy relation for optical transitions as a function on the number of the atoms in each NCs, *E*_*Fermi*_/*N*^1/3^, we obtain an expression for the PL intensity by transforming Eq. 1 to the energy axis^50^:

2 $${I}_{PL}=A{(\frac{q}{{E}_{PL}})}^{3-\gamma }{e}^{-\frac{{(\frac{q}{{E}_{PL}}-\langle D\rangle )}^{2}}{2{\sigma }^{2}}}$$

where *q* = 2*r*_*w*_*E*_*f*_, *E*_*PL*_ is the emission energy and *A* is an arbitrary constant. In Fig. 4(a) we simulated a PL curve for Au NCs and several gaussian distributions with mean size from 0.9 nm to 1.9 nm. The parameter was chosen to be *γ* = 11 in order to correctly describe the quenching of PL emission for NCs with sizes near to 2 nm. In the case of Pt NCs, the chosen parameter is *γ* = 9 for the same reason. The simulated PL using Eq. 2 is shown in Fig. 4(b,c,d) and the fitting parameters 〈*D*〉 and *σ* are also shown in each case. In particular for AgNCs in sapphire, the sum of two Gaussian distributions for the NC size was used. The fitting is fairly good and the mean size and standard deviation coincides very well with those discussed in Table 1. As a conclusion we can state that peak PL emissions seems to be determined by the bulk Fermi energy of the metal and their Wigner Seitz radius. This result means that the photophysic of metal NCs embedded in silica or sapphire matrices can be described by the free electron Jellium model. An interesting result is that *γ* parameter for the oscillator strength is different for Pt NCs. This argument can be used to explain why it is possible to observe larger Pt NCs with more intense PL compared with Ag or Au NCs.

**Figure 5:**
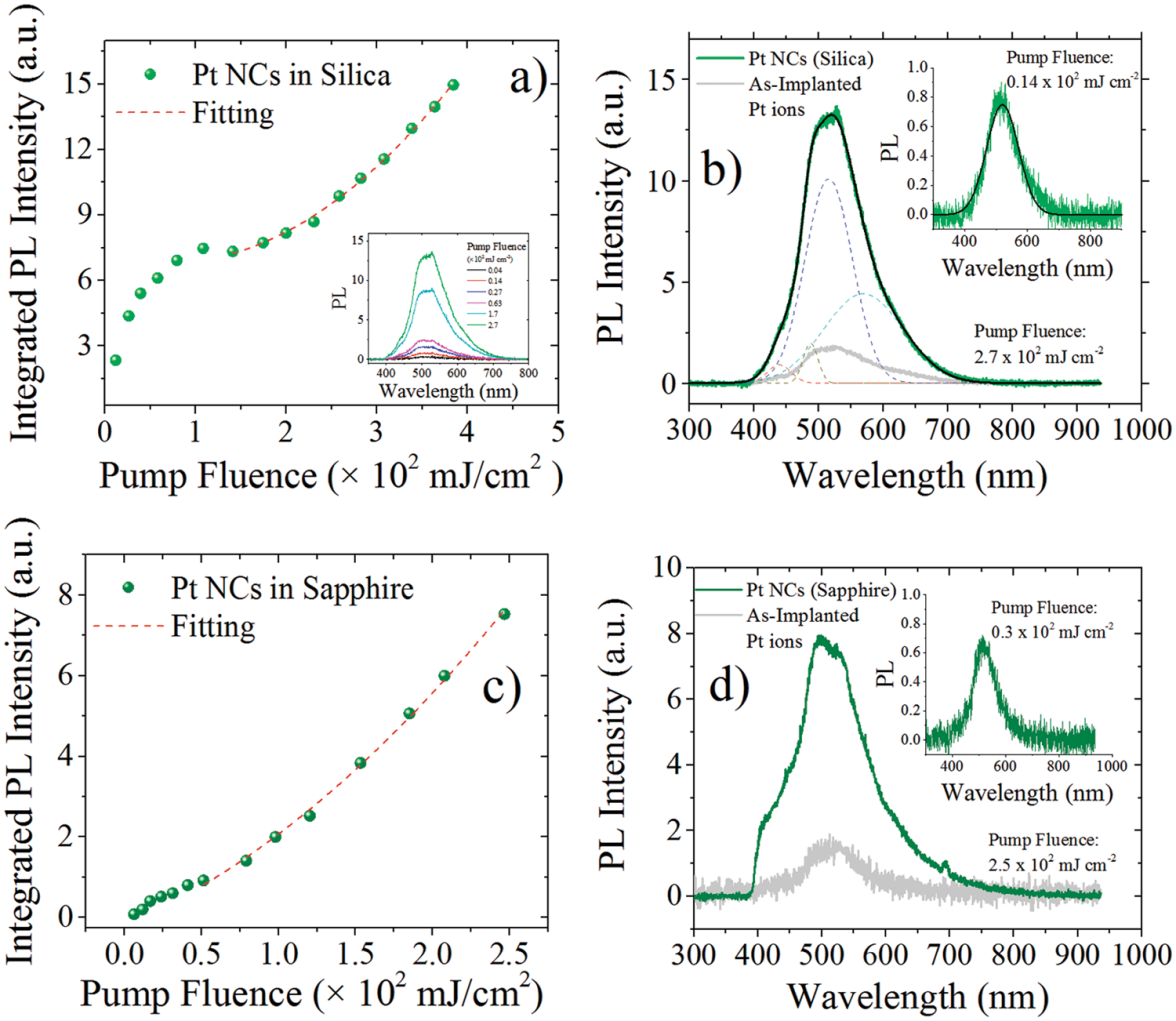
Integrated PL intensity vs Pump laser excitation fluence at 355 nm for Pt NCs: (**a**) in silica; (**c**) in sapphire. Inset in (**a**) shows the PL spectra for different pump fluences. PL spectra at high and low pump laser excitation (inset graph) for Pt NCs: (**b**) in silica; (**d**) in sapphire. The PL emission corresponding to the as-implanted sample, without thermal annealing, is also shown (gray curve). Deconvolution of the PL spectrum in (**b**) is also shown (dashes curves). Data in each graph were normalized to be comparable.

**Figure 6:**
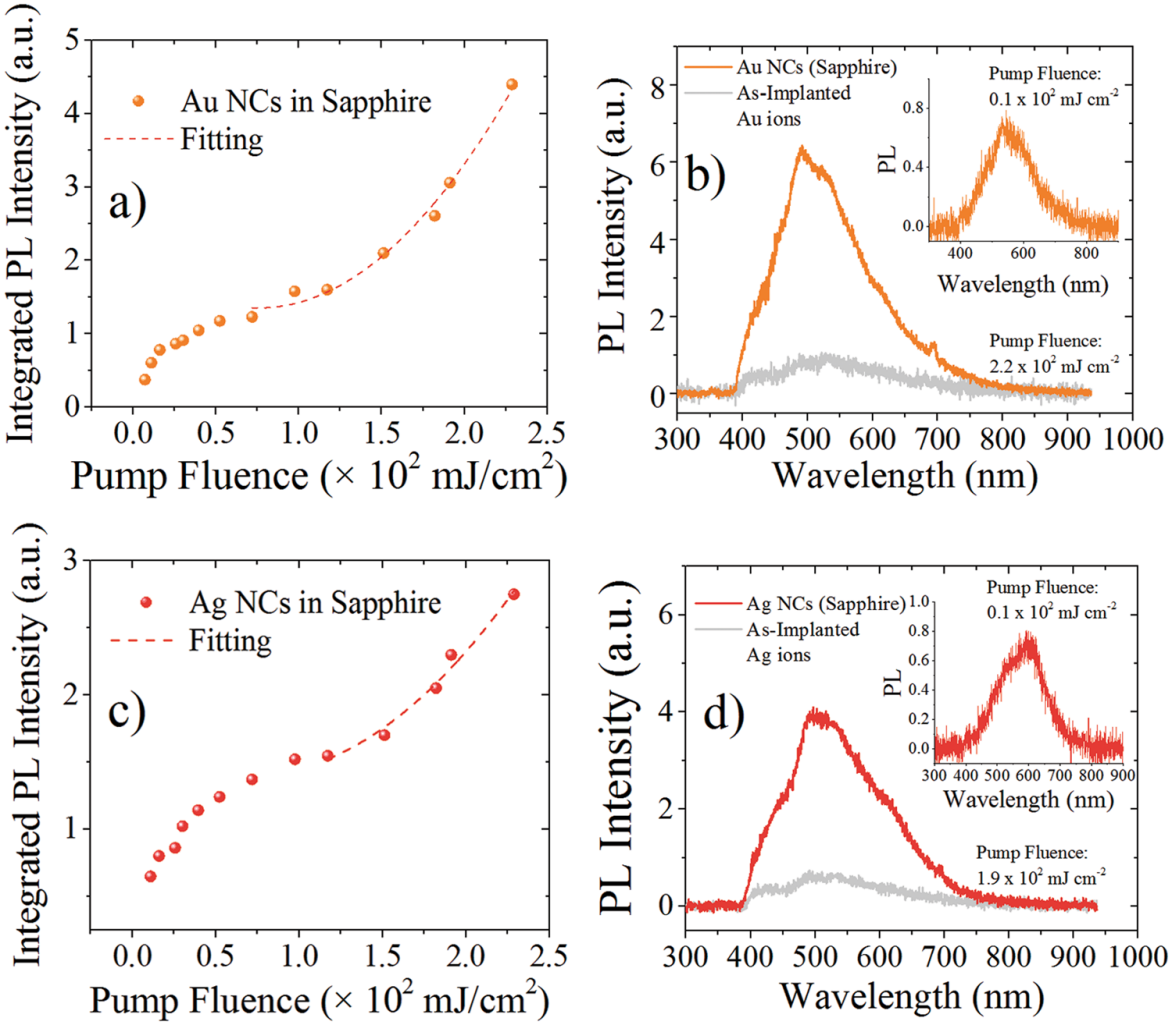
Integrated PL intensity vs Pump laser excitation fluence at 355 nm for: (**a**) Au and (**c**) Ag NCs in sapphire. PL spectra at high and low pump laser excitation (inset graph) for: (**b**) Au and (**d**) Ag NCs. The PL emission corresponding to as-implanted sample, without thermal annealing, is also shown (gray curve).

Finally, we find that PL emission from these metal NCs are very stable under picosecond laser excitation at 355 nm, and no degradation of their PL intensity was observed even with an excitation of 8 mJ per pulse with a spot size of 1–2 mm. Moreover, we explored the PL intensity vs pump laser excitation at 355 nm and results are shown in Figs 5 and 6. Integrated PL intensity in Figs 5 and 6 was obtained as the area under the experimental PL curve for each pump fluence excitation. As it can be seen from Figs 5 and 6, the initial part of the PL intensity vs pump power is linear and tends to be saturated as the irradiance increases. At higher power pump excitation, the PL intensity takes an upward turn and increases superlinearly. The increase of PL scales quadratically with the pump fluence, ${I}_{PL} \sim {P}_{exc}^{2}$, as shown by the fitting curve in each panel graphs. It is well known that semiconductor quantum dots exhibit a PL linear increase with pump fluence and saturate at higher fluences due to a very efficient Auger desexcitation or the presence of surface state that can trap excitons.

Superlinear PL response have been observed recently in some nanoemitters such as single quantum dots^37^, Zn-doped GaN^51^, and in plasmon-quantum dots coupling systems^52,53^. Below room temperature, quantum structured semiconductors also exhibit a superlinear PL behavior^39,54–57^. Moreover, studies of PL from phosphor^58^ and photoconductive phosphor^59^ showed a superlinear increase in PL intensity. A quadratic increase of PL intensity have been also observed in quantum dots which exhibit PL emission by two-photon absorption under ultrafast laser excitations^40,60^. In general, the origin of the superlinear PL behavior is closely related to the dynamics of the photoexcited carriers in the nanostructure. At lower intensities one electron-hole pair is created inside the NCs emitters. Then radiative recombination of the *e-h* pair gives rise to PL emissions. When pump excitation increases, the probability of getting two or more *e-h* pairs also increases and Coulomb interaction between carriers can result in nonradiative emission; therefore, saturation of PL intensity could be observed. However, if Auger recombination is suppressed the PL does not saturate and more than one pair of *e-h* per NCs can generate photon emission through an efficient radiative transition^37,39,51,54,56^. If the photogenerated *e-h* pairs remain bound due to their Coulomb interaction, they can form an exciton^61^ (or a biexciton state for two *e-h* pairs^36^) and then they can be used as a laser media^62^. On the other hand, the photogenerated *e-h* pairs can be dissociated into free carriers (unbound electrons and holes), a valuable mechanism for photovoltaic and solar cell devices^62,63^. The superlinear PL observed below room temperature in quantum structured semiconductors has been attributed to uncorrelated *e-h* pairs photogenerated carriers^38,39,57^ or due to the saturation of temperature activated trap-state^54,56^. Superlinear PL from single quantum dots and plasmon-quantum dots coupling system have been ascribed to biexciton states^37,52,55^. For single semiconductor NCs, if the Auger recombination rate is low, new emissions peaks can emerge in the PL spectrum once the exciton state starts to be populated. In case of biexciton effects, new emission peaks can occur at higher or lower energies than those observed for single exciton emissions.

**Figure 7:**
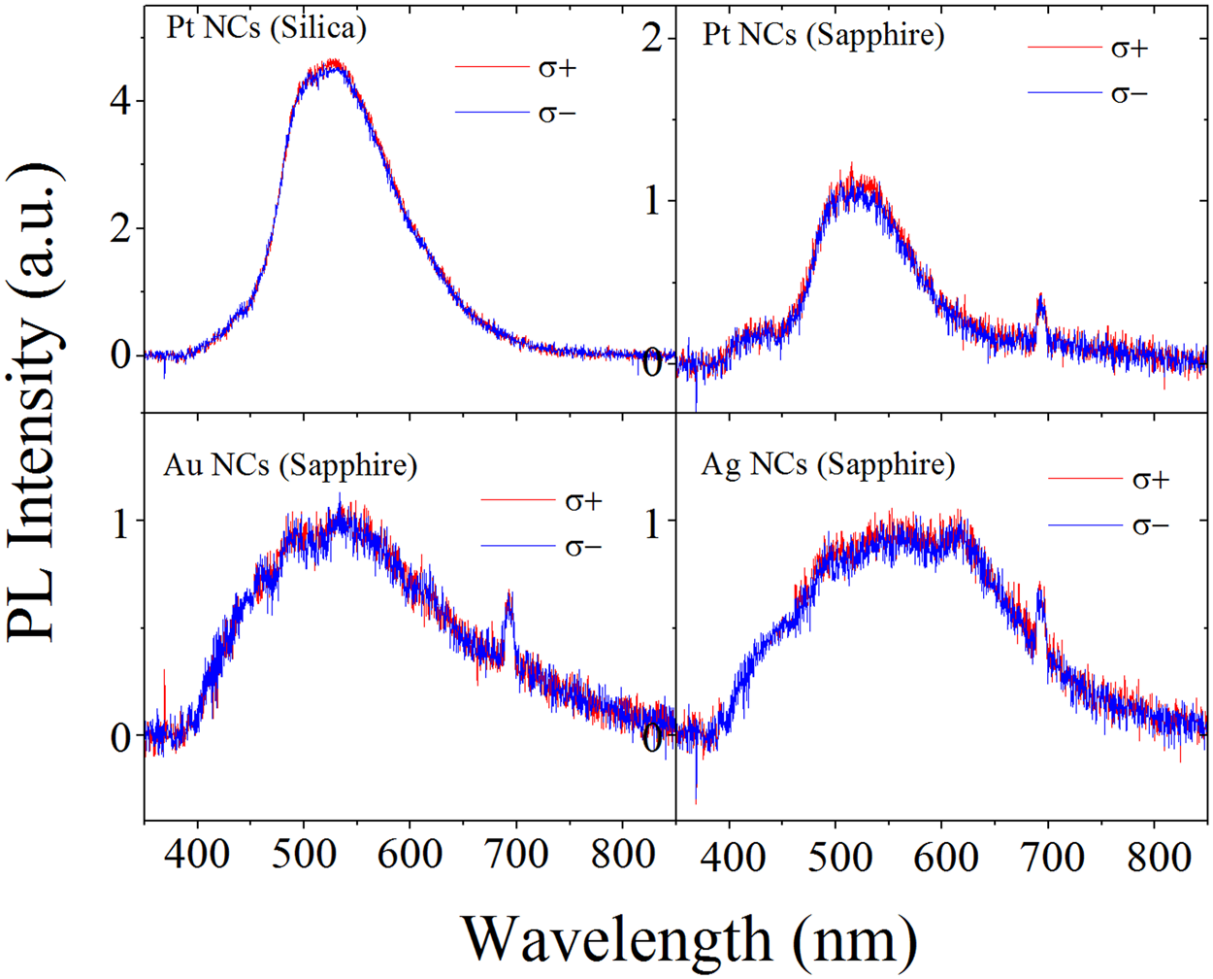
PL spectra of Pt NCs in silica and Ag, Au and Pt in sapphire by using right (*σ*+) and left (σ−) circularly polarized light. The pump laser fluence was around 180 mJ/cm^2^.

Excitons in metal have not been clearly observed in experiments because of the screening effects, taking place at femtosecond timescales, even though they have been theoretically considered. Excitonic properties have been calculated by ab-initio models for ultrasmall Ag cluster^64^. Recently it has been reported evidence of a transient excitonic response on a silver surface^65^ and excitonic properties have been measured in ultrasmall Au clusters^64^. In our studies the emission provided by individual or isolate metal NCs was not considered and instead integrated PL response was analyzed. The PL spectra measured at high pump laser excitation for the samples studied in this work is shown in Figs 5 and 6. The PL spectrum for Pt NCs in silica at high power excitation was deconvoluted and several peaks are shown in Fig. 5(b). In contrast, the PL spectrum at low power excitation shown in the inset plotted in Fig. 5(b), cannot be deconvoluted in these peaks and only one peak is required in order to describe the PL curve. On the other hand, at high power excitation the PL spectrum of Pt NCs in sapphire is also different from the one observed under low power excitation (inset in Fig. 5(d), and then it can be decomposed in several new peaks (not shown). Similar results can be observed in Fig. 6(b,d) for Au and Ag NCs, respectively. A fingerprint of biexciton emission can be obtained by using right (*σ*+) and left (*σ*−) circularly polarized beam laser excitation^55,61,64^. However, when using circularly polarized light, the samples studied in this work did not exhibit any significant change in spectral PL emission, as shown in Fig. 7. Then the superlinear behavior cannot been unambigously adscribed to biexciton emissions, and instead the carrier dynamic is probably dominated by uncorrelated electrons and holes pairs. A quadratic pump intensity dependence of PL signal can be also an indication of a two-photon excitation process in which higher energy states can be achieved by a two-photon absorption at 532 nm. Further investigations using transient absorption spectroscopy or time resolved measurements can give more information about the actual dynamics of the carrier in the excited state of the embedded metal NCs.

It is important to notice that the pump fluence at which superlinear PL behavior starts is about 150 mJ/cm^2^ for Pt NCs in silica while for those in sapphire is significatively lower about 50 mJ/cm^2^. In the case of Au and Ag NCs in sapphire the threshold for superlinear PL is about 70 mJ/cm^2^ and 120 mJ/cm^2^, respectively. The lower threshold fluence for superlinear PL behavior in Pt NCs in sapphire compared with that in silica can be related to the presence of larger plasmonic metal NPs in sapphire (see Fig. 1(a,b). The plasmon resonance for Pt NPs is located in the UV spectral range and the excitation laser is at 355 nm within this resonance absorption band. Plasmonic Pt NPs near Pt NCs inside the sapphire matrix can influence the photophysics of superlinear emission by plasmon coupling^52^.

In order to discard the influence of ion-implantation damage in the origin of the new spectral characteristics and the superlinear behavior under high power excitation, we measured the PL spectrum of the as-implanted samples, i.e. samples implanted with metal ions but without thermal annealing and results are shown in gray PL curves in Figs 5(b,d) and 6(b,d). As we can see the PL intensity for these samples are much less intense than those observed in annealed samples. This weak emission can be attributed to ultra-small clusters formed in the matrix during the ion bombardment of the sample. The ion-implantation performed in this work was carried out at room temperature, however, thermal heating due to ion collisions close to 100 °C can be expected and this temperature could promote some early stage for metal NC nucleation. It is important to notice that if the observed PL emission of our sample would come from matrix defect state as a result of ion-implantation damage, then the as-implanted sample could have exhibited higher PL intensities compared to those observed in the annealed samples, where the defects can be passivated and matrix is recovered from damage. Moreover, in our experimental studies we used an excitation wavelength of 355 nm and at this wavelength silica defect states cannot efficiently absorb or emit light because the excitation wavelength is far from the corresponding absorption peak in silica, as we have pointed out in a previous work^41^. Defect states in sapphire are also well studied and most of them have their absorption band below 330 nm^66,67^. F_3_^+^ color center in sapphire has an absorption band peaked in 361 nm, but its PL emission occurs at 380 nm^66^, out of the range where we observed the PL emission for the samples studied in this work. Aditionally, we implanted metal ions with similar atomic masses, which should result in similar defect concentrations in sapphire, then leading to a comparable PL response. However, our results showed PL spectra peaked at different wavelengths and with remarkable different intensities, depending on the implanted metal ions and thermal treatments. Taking into account the PL spectral differences observed in the sample whose peak emissions scale according to the Jellium model energy law, and the presence of ultra-small metal NCs as revealed by TEM observation and optical absorptions measurments, we are confident that the observed emission comes univocally from metal NCs embedded in the matrices. Further studies to experimentally determine the exact size distributions of metal NCs in sapphire must be done, and can contibute to fully understand the relation between NCs size and energy emissions.

## Methods

### Sample preparation

2 MeV Ag, Au and Pt ions were implanted at room temperature with a fluence of ~2.5 × 10^16^ *cm*^−2^ in high-purity sapphire plates using the 3 MV Tandem accelerator (NEC 9SDH-2 Pelletron) at IFUNAM (Instituto de Física, Universidad Nacional Autónoma de México). With this fluence is possible to obtain enough implanted material to observe a significant plasmon absorption band and PL emission intensities in our experiments to confirm the nucleation of metal nanoparticles^35^. After metal ion-implantation in sapphire each sample was heated under a reducing atmosphere (RA) compound of 50%N_2_ + 50%H_2_ for 90 minutes at a temperature of 950 °C for samples with Au and Ag ions and 1050 °C in the case of Pt ions. Annealing temperatures above 800 °C allow the complete passivation of optically active defects and the damage recovery in sapphire^35^. Pt NPs were also synthesized in high-purity silica plates by means of a 2 MeV ion beam with a fluence of ~2.5 × 10^16^ *cm*^−2^, followed by an annealing at 600 °C for 60 minutes under RA. This thermal treatment promotes the sample damage recovery to obtain a defect free ion-implanted silica matrix^27,41^. The implanted ion fluences and the concentration depth profiles were determined by Rutherford Backscattering Spectrometry (RBS), with a 2 MeV ^4^He^++^ beam. The Pt ions implanted in silica have a projected range (*R*_*P*_) of 580 nm with a full width half maximum (FWHM) of 284 nm. Data on ${R}_{P}$ and FWHM for the different implanted ions in sapphire are shown in Table 2.

**Table 2:** Ion implanted distribution in sapphire: Peak of depth under the substrate surface (*R*_*P*_) and their FWHM.

Implanted Ions	Sapphire Matrix	
	*R*_*P*_ (nm)	*FWHM* (nm)
Pt	335	186
Au	329	187
Ag	520	251

### Optical characterization

PL measurements were conducted by using picosecond pulsed Nd:YAG laser (EKSPLA) excitation with a vertically polarized beam at 355 nm, a frequency repetition rate of 10 Hz and an energy per pulse about 150 *μ*J. Excitation was performed at normal incidence with a spot size of about 3–4 mm. PL was detected at 45° from the normal surface of sapphire or silica plate at the same side of the laser incidence beam. In order to avoid any possible experimental disturbance when measuring with the 355 nm beam, the experimental setup must filter out the second harmonic beam at 532 nm, which usually comes out along with the 355 nm beam. Therefore, we used 355 nm mirrors to reduce the reflection of the beam at 532 nm and a vertical linear polarizing cube to eliminate the 532 nm signal, which is horizontally polarized. By using a half-wave plate, we also observed that there is no influence of incident beam polarization on PL intensity. Right and left circularly polarized excitation at 355 nm was performed by using a quarter-wave plate. Photostability under strong laser excitation was observed at even higher laser energies per pulse up to 8 mJ and no optical damage neither degradation of their PL intensities were noticed. Emitted light was focused by a broadband convergence lens, 2.5 cm of diameter and a focal length of 10 cm, on an optical fiber connected to an OCEAN optics (USB 2000+) spectrometer. A long band-pass filter (400 nm) was used to block any reflected or scattered beams from incidence laser excitation. Photoluminescent intensity vs pump fluence was measured by using a spot diameter of 1.5 or 2 mm and energies per pulse in the 50 *μ*J to 8 mJ range. High pump excitation experiments were performed at a laser irradiance below the ablation threshold for the silica and sapphire matrices: ∼500 mJ/cm^2^ and ∼700 mJ/cm^2^, respectively. Optical absorption spectra were measurement with a UV-Vis Varian Cary 5000 spectrophotometer. Results were corroborated by undertaking several optical experiments at different areas of the same sample with nucleated metal particles. Also, at least a second set of samples was synthesized under similar conditions and the corresponding results are consistent with the data reported in this work. Optical emission and spectral characteristics remained unchanged at least for the 2-years period devoted to this research. TEM micrographs in silica samples were obtained by using a FEI Tecnai F30 transmission electron microscope (FEG-TEM 300 kV) in Bright Field Mode.

## Conclusions

Bright visible photoluminescent from sub-nanometer-sized (<2 nm) metal NCs (Pt, Ag, Au) prepared by ion-implantation and embedded in sapphire plates is reported. Intense photoluminescent emission was also observed for Pt NCs implanted in silica matrix, sixfold larger than the one exhibited by Pt NCs in sapphire. Synthesis condition used in this work allows for the nucleation of sub-nanometer-sized metal clusters that give rise to interesting photoluminescent properties. Pt NCs in silica or sapphire matrices display similar spectral characteristics under low pump power excitation (<50 mJ/cm^2^): PL peak at 530 nm and a spectral width of 100 nm. Similar spectral characteristics suggest that photon emission is coming from optical transitions in the core of the Pt NCs and that the matrix host do not affect the optical transitions. The difference observed in PL intensity for Pt NCs in sapphire and silica is directly related to the number of emitters in each sample. Optical absorption measurements reveal that in sapphire matrices, a large number of plasmonic particles can be obtained and in consequence a minor quantity of photoluminescent Pt NCs compared to those in silica. Jellium law scaling relation, *E*_*Fermi*_/*N*^1/3^, was used to numerically simulate the energy emission spectra for each metal NCs. The PL spectra for the different NCs were theoretically simulated using the bulk Fermi energy of each metal and a Gaussian size distribution for the NCs. Our results indicate that the optical emission studied is derived from quantum confinement effect.

Photoluminescence from Pt (green), Ag (orange), Au (yellow-orange) NCs do not show degradation in their spectral characteristic under high-energy picosecond pulsed laser excitation. Furthermore interesting superlinear PL was observed under high pump fluence excitation (>100 mJ/cm^2^). A quadratically increase of PL signal with pump pulse fluence and the presence of new PL peaks in deconvoluted PL spectrum was observed. Circularly polarized light measurements did not reveal any change in the spectral characteristics of the sample at higher power excitation. This behavior indicates that the carrier photodymanics under high power excitation is dominated by uncorrelated electron-hole pairs. The minimum pump fluence excitation to observe a superlinear PL increase is different in each sample: 150 mJ/cm^2^ and 50 mJ/cm^2^ for Pt NCs in silica and sapphire, respectively, 70 mJ/cm^2^ for Au NCs and 120 mJ/cm^2^ for Ag NCs in sapphire. The lower threshold pump fluence associated with the starting of the superlinear PL behavior for Pt NCs in sapphire is related to the presence of larger plasmonics NPs nucleated after the thermal treatment, which are most abundant in the sapphire sample and coexist with metal NCs. Further investigation of the effects of pump fluence, transient absorption spectroscopy etc., should clarify the phenomenology, and ultimately lead to a better understanding of the microscopic carrier relaxation mechanisms associated with metal NCs. Besides, potential applications in nanoscale solid state white light emitters can be addressed with this nanocomposite by combining different metal clusters such as Pt and Ag, whose emission spectra cover the entire visible spectrum and their sub-nanometer size is valuable for further demanding miniaturization in nanoscale optoelectronic devices.

## Data Availability

The data that support the findings of this study are available from the corresponding author, J.B., upon reasonable request.
